# ChemDes: an integrated web-based platform for molecular descriptor and fingerprint computation

**DOI:** 10.1186/s13321-015-0109-z

**Published:** 2015-12-09

**Authors:** Jie Dong, Dong-Sheng Cao, Hong-Yu Miao, Shao Liu, Bai-Chuan Deng, Yong-Huan Yun, Ning-Ning Wang, Ai-Ping Lu, Wen-Bin Zeng, Alex F. Chen

**Affiliations:** School of Pharmaceutical Sciences, Central South University, Changsha, 410013 Hunan People’s Republic of China; Department of Biostatistics, School of Public Health, University of Texas Health Science Center at Houston, Houston, USA; Xiangya Hospital, Central South University, Changsha, 410008 Hunan People’s Republic of China; College of Chemistry and Chemical Engineering, Central South University, Changsha, 410083 People’s Republic of China; Institute of Advancing Translational Medicine in Bone & Joint Diseases, School of Chinese Medicine, Hong Kong Baptist University, Kowloon Tong, Hong Kong SAR People’s Republic of China

**Keywords:** Molecular descriptors, Molecular fingerprints, Online descriptor calculation, QSAR/QSPR, Molecular representation, Chemoinformatics

## Abstract

**Background:**

Molecular descriptors and fingerprints have been routinely used in QSAR/SAR analysis, virtual drug screening, compound search/ranking, drug ADME/T prediction and other drug discovery processes. Since the calculation of such quantitative representations of molecules may require substantial computational skills and efforts, several tools have been previously developed to make an attempt to ease the process. However, there are still several hurdles for users to overcome to fully harness the power of these tools. First, most of the tools are distributed as standalone software or packages that require necessary configuration or programming efforts of users. Second, many of the tools can only calculate a subset of molecular descriptors, and the results from multiple tools need to be manually merged to generate a comprehensive set of descriptors. Third, some packages only provide application programming interfaces and are implemented in different computer languages, which pose additional challenges to the integration of these tools.

**Results:**

A freely available web-based platform, named ChemDes, is developed in this study. It integrates multiple state-of-the-art packages (i.e., Pybel, CDK, RDKit, BlueDesc, Chemopy, PaDEL and jCompoundMapper) for computing molecular descriptors and fingerprints. ChemDes not only provides friendly web interfaces to relieve users from burdensome programming work, but also offers three useful and convenient auxiliary tools for format converting, MOPAC optimization and fingerprint similarity calculation. Currently, ChemDes has the capability of computing 3679 molecular descriptors and 59 types of molecular fingerprints.

**Conclusion:**

ChemDes provides users an integrated and friendly tool to calculate various molecular descriptors and fingerprints. It is freely available at http://www.scbdd.com/chemdes. The source code of the project is also available as a supplementary file.

**Graphical abstract:**
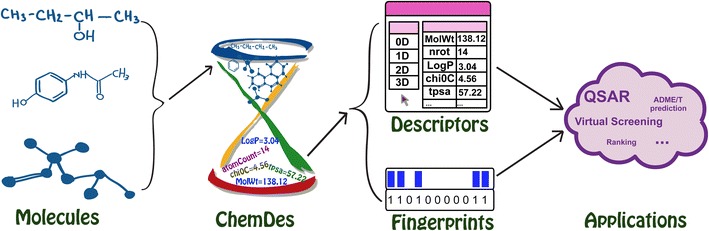
An overview of ChemDes. A platform for computing various molecular descriptors and fingerprints

## Background

Molecular descriptors are experimentally-measured or theoretically-derived properties of a molecule [1]. More specifically, they are quantitative representations of physical, chemical or topological characteristics of molecules that summarize our knowledge and understanding of molecular structure and activity from different aspects. Molecular fingerprints are property profiles of a molecule, usually in forms of bit or count vectors with the vector elements indicating the existence or the frequencies of certain properties, respectively. Both molecular descriptors and fingerprints play a fundamental role in QSAR/SAR analysis, virtual molecule screening, similarity-based compound search, target molecule ranking, drug ADME/T prediction and the other drug discovery processes [2–12].

Various molecular descriptors and fingerprints have been developed in previous studies for quantitative molecular representation. Besides their extensive usage in the aforementioned regular applications (e.g., QSAR/QSPR modeling based on machine learning techniques [13–16]), molecular descriptors and fingerprints are also shown to have a significant potential to play a critical role in studies of current scientific interests, such as the identification of biomolecular targets and the network analysis of protein–ligand interactions. For example, Bork et al. [17] successfully identified certain potential targets by combining the chemical similarity and side-effect similarity. Keiser et al. [18] investigated the relationships between protein function similarity and ligand structure similarity to predict new high-potential drug targets. Furthermore, several studies employed molecular descriptors or fingerprints to predict drug-target interactions or understand the action mechanisms of drugs [19–23]. In addition, molecular descriptors or fingerprints were also used to characterize the structural information of amino acids or nucleotides for developing more effective protein or RNA/DNA descriptors [24–26].

Existing tools for molecular descriptor and fingerprint calculation include DRAGON [27], BlueDesc [28], CDK Descriptor Calculator [29], PaDEL [30], Mold2 [31], ChemAxon JChem [32], ADMEWORKS ModelBuilder [33], CDK [34], RDKit [35], Chemopy [36], etc. Several generic drug design software such as MOE [37], SYBYL-X [38] and Discovery Studio [39] also provide the descriptor calculation functionalities. However, many of these tools only covers a subset of molecular descriptors and/or fingerprints such that users need to manually merge the outcomes from multiple tools to obtain a comprehensive set of results, which inevitably take a certain degree of unnecessary and tedious efforts. Also, as standalone packages, the deployment of these tools may require users to go through a sophisticated installation and configuration process, which could be challenging for entry-level users. More importantly, some of the tools mentioned above (e.g., RDKit) only provide application programming interfaces to users and different tools are implemented in different computer languages, which significantly hamper the broader applications of these tools. It is therefore useful to integrate and provide these tools to end users in a more friendly way.

In this study, we developed a freely-available web-based platform called ChemDes, which provides an online service to the public for calculating a variety of molecular descriptors and fingerprints conveniently and instantly. More specifically, ChemDes can compute 3679 descriptors and 59 types of molecular fingerprints, including, e.g., the one-dimensional bulk properties of compounds, the two-dimensional topological and charge indices, and more complex three-dimensional (3-D) descriptors. Additionally, ChemDes provides three useful auxiliary tools, named ChemCONV, ChemMOP and ChemFPS, for convenient format converting, MOPAC optimization and fingerprint similarity calculation, respectively. We thus believe ChemDes is a useful platform that better suits the needs in related chemoinformatics and bioinformatics studies.

## Implementation

### Development environment

Python programming language has been becoming very active in the research community because of its scalability and rich library functions. In ChemDes, Python is chosen as the main development language because it could work well with other tools or packages developed by different programming languages, and they have good interaction and compatibility with each other. There are also plenty of libraries for the scientific computation such as *Numpy*, *scikit*-*learn* and *Pandas*. Moreover, some packages or tools used in ChemDes such as Pybel and RDKit all provide the Python application program interfaces (APIs). This makes it possible to integrate these different resources in the Python language framework.

The whole system runs on an ECS (elastic compute service) server of Aliyun. The number of CPU cores and memory are automatically allocated to the running instances on demand, which ensures the elastically stretchable computing capability. Django is chosen as a high-level Python web framework to encourage the rapid development and clear design. According to its model-visualization-control (MVC) design pattern, the whole system is divided into three main components: the back-end calculating program, the back-end control program and the front-end visualization program. At the back-end, the authors use uWSGI + Nginx as the web server, and use MySQL database for data storage and retrieval. At the front end, the website is designed in accordance with W3C standards. In addition, the JavaScript and jQuery are also utilized to accomplish some complex interaction processes which could effectively avoid potential problems of some strict runtime environment and security risks.

### Input/output system

The Input/output system, as the basic part of the ChemDes platform, is mainly responsible for the input or output of the strings, commands and files. ChemDes uses the functions like *input, file, open, write, getcwd* and *setcwd* from Python I/O system to accomplish the file reads and writes. In order to rapidly handle large files, the other methods like *Chunks* are used to make sure the success of uploading and storing large files. In addition, relative paths instead of absolute paths are also used to enhance the transportability of the platform. Herein, ChemDes only accepts molecular file types in the formats of *SMILES* and *SDF*. However, before transferred to the back-end calculating program, these inputs must be validated by the format authentication and the format validation programs. Moreover, the *SDF* format input will accept an extra validation of format legitimacy through a piece of JavaScript program at the front end. After the structure identification, molecules will be parsed and instantiated as different molecular objects such as *pybel.Molecule, rdk.Molecule (myMol)* and *Molecule (myCDKMolecule)*. Different molecular objects have different attributes and methods, which is the main premise of calling the corresponding calculating functions. Herein, the authors integrated the corresponding molecular wrappers and realized the molecular information exchange between different molecular objects. Once these molecular objects are successfully obtained, the back end calculating program could call them to calculate the corresponding molecular descriptors.

To facilitate the user’s application to the ChemDes platform, a useful auxiliary tool called ChemCONV was developed to realize the format conversion between dozens formats of molecular files. ChemCONV allows users to import 7 types of formats and export 11 types for extensive applications, and it also realized the batch computing by submitting a molecular file with multiple molecules. We suggest that all formats of molecular files should be firstly converted to *SMILES or SDF* in these situations. This will be an effective way to avoid the exception caused by these situations.

### MOPAC optimization

When 3-D molecular descriptors are calculated, chemical structures should be optimized in advance to obtain 3-D coordinates or atom charge information. Herein, the authors choose MOPAC [40] to accomplish this work. MOPAC is a general-purpose semi-empirical molecular orbital package. Molecular optimization driven by MOPAC is widely employed to optimize the molecular structure in QSAR/QSPR and the other applications in chemoinformatics. Compared with the other molecular optimization programs, MOPAC includes more built-in molecular force fields, which will give us multiple choices to perform the optimization and reduce the risks that may arise from a single method. Consequently, ChemDes provides seven semi-empirical methods for the molecular optimization, including AM1,PM3, MNDO, MNDO-d, RM1, PM6, and PM7 [41, 42]. Users can choose one particular molecular force field to perform the optimization according to their needs. Additionally, it will be less time consuming than the other traditional ab initio optimizing method such as Gaussian program. This is very important for the computation of 3-D molecular descriptors, especially for an instant computing platform. It should be noted that the MOPAC optimization module will only be activated when users submit a job to compute 3-D molecular descriptors. Additionally, a full-time molecular optimization module called ChemMOP was also developed to perform the molecular optimization operation conveniently.

### Integration of APIs

**Fig. 1:**
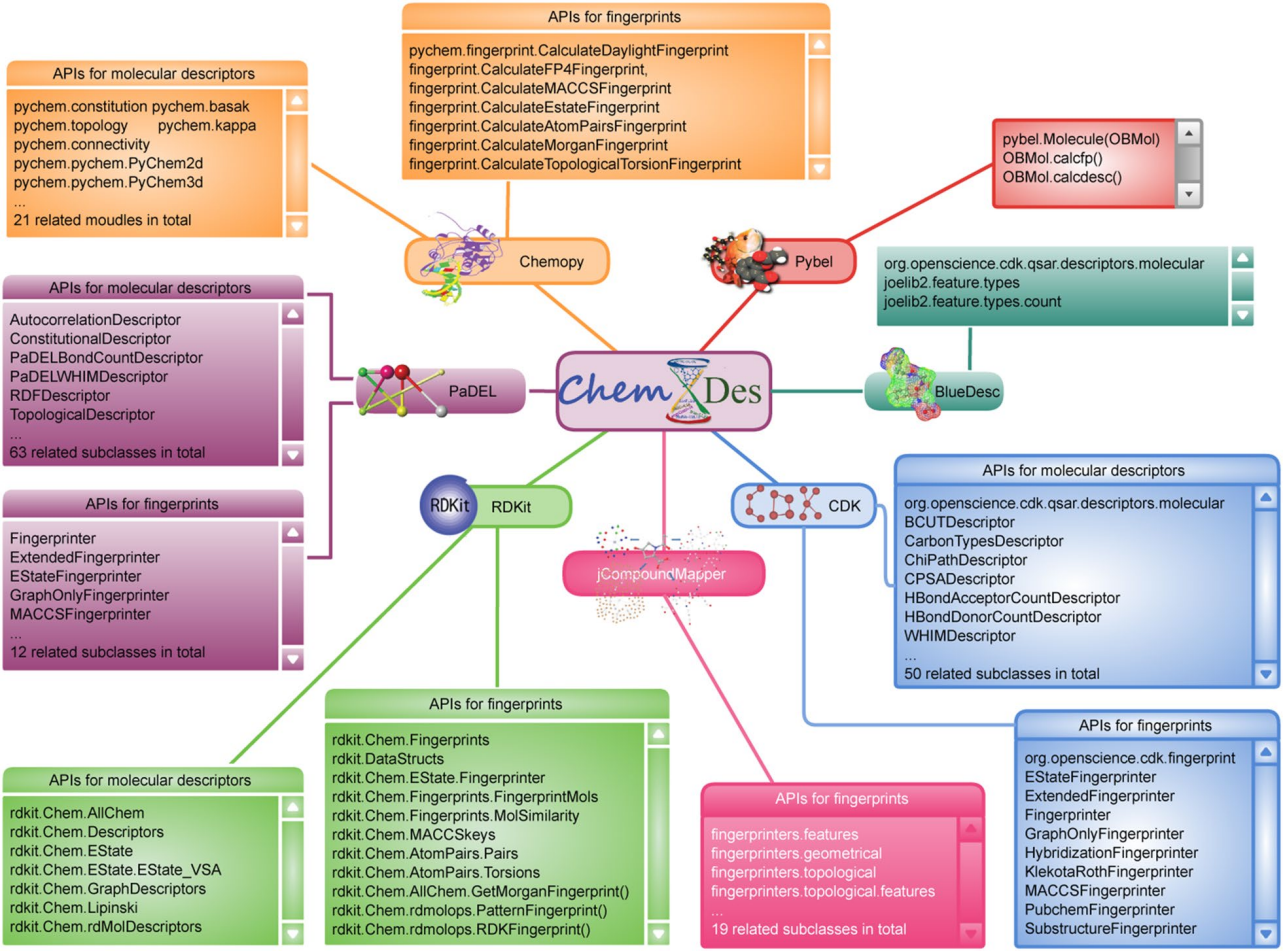
The APIs integrated in ChemDes. ChemDes integrated all the APIs related to molecular descriptors and fingerprints from six toolkits. The APIs from each toolkit are divided into two main parts: the APIs for molecular descriptors and the APIs for fingerprints

ChemDes integrates seven toolkits to calculate a large number of molecular descriptors and fingerprints, including Pybel [43], RDKit, CDK, Chemopy, BlueDesc PaDEL, jCompoundMapper [44]. To perform these operations, we need to integrate all the related APIs in the back-end calculating program. A brief list of these APIs is summarized in Fig. 1. Because these seven toolkits are written and called in different program languages, it is very difficult to integrate them into a unified platform. However, ChemDes integrates various APIs in different program languages. Some packages such as Chemopy, Pyebl and RDKit are written in Python language or have support for Python script. In other words, they all have ready-for-use Python APIs. Take RDKit for an example, we choose its 7 functionalities related with molecular descriptors and 11 functionalities related with molecular fingerprints. However, these functionalities have different specifications and implementations. Some of these functionalities are rooted in the modules and some are callable functions; in some cases, the functions require default parameters; different functions may generate different object types; the results of fingerprints of count version contain frequency information while the bit version does not. In order to realize the efficient and unified computation, particular modules with corresponding classes or functions have been developed to handle these situations. A *calc_fun* module is developed to reorganize the related APIs and assure a unified way to call the functions. It also responds to work out the corresponding descriptor names and their types. For example, it picks out the functions like *CalcChi0n*, *CalcChi0v* and *CalcChi1n* from the *rdkit.rdMolDescriptors* and puts them into connectivity descriptors, the functions like *CalcNumRings*, *CalcNumAmideBonds* and *CalcNumRotatableBonds* into constitutional descriptors. A *result_wash* module is also developed to deal with different results and to allow for uniform access to the descriptor values via the methods from the alternative logical branches. Obviously, it is a barrier to interoperability that one programming language usually cannot simultaneously access more than one toolkit. Among these toolkits, CDK, BlueDesc and PaDEL are Java software and do not have exactly APIs for Python. In order to access Java libraries from CPython, the Python library *JPype* is needed. This starts an instance of the Java virtual machine and uses the Java native interface to communicate back and forth. By using *JPype*, we are allowed to obtain the Java class or call the Java functions related to the APIs listed here.

### Multitasking server architectures

A web-based platform must have robust multitasking architectures to enable different users to obtain services at the same time. To meet this need, the Nginx + uWSGI architecture is used. We use the preforking operational mode of uWSGI with the multiply interpreters. uWSGI serves responses to the Nginx via the WSGI protocol. The dynamic data from interaction between Python computational program and uWSGI will then interact with Nginx, and the latter will serves results to the clients in form of static contents. Additionally, in order to meet time requirement for data operation, the authors have optimized some related parameters of Nginx and uWSGI such as *max*-*requests*, *harakiri* and *keepalive_timeout*. By employing the certain architectures, the balance between system resource occupation and computational efficiency is maintained; the good independence and safety of a long time data operation and file access from different requests are also guaranteed.

## Results

### User interface

To provide an online computing service based on web, the user interface should be convenient and easy-to-use for the users. Herein, the user interface of ChemDes consists of four main modules: “Webserver”, “Library”, “Tools” and “Help”. The “Webserver” is the main entrance for users to calculate molecular descriptors. It provides different entrances according to the sources and types of molecular descriptors. The “Library” module provides the detailed definitions and references for all molecular descriptors and fingerprints that can be calculated by ChemDes. It would be very convenient to check and interpret the meaning of each molecular descriptor. The “Tools” module provides the entrance for the three auxiliary tools (ChemCONV, ChemMOP and ChemFPS). These three useful auxiliary tools help the users conveniently perform format converting, MOPAC optimization and fingerprint similarity, respectively. At last, the “Help” module provides detailed instructions of all the major functions of this platform, and some frequently asked questions and the solutions are also listed there. The users could also ask more questions and provide some suggestions to help us improve the ChemDes platform. In addition to the four main parts mentioned above, there are also some other functions that will not be described in details here. For example, the functions of structural examination and visualization from JSDraw [45]. These functions may be triggered in related stages, and then finish their missions.

### Computation of molecular descriptors

ChemDes computes 1-D/2-D descriptors representing molecular properties and structural information from the molecular graph. Most of them have garnered considerable interest because of the ease of generation and the fast speed with which these computations can be completed. They have been extensively used in modeling various physicochemical properties as well as biological activities. Currently, ChemDes allows users to compute 2618 1-D/2-D molecular descriptors conveniently. As we know, 1-D/2-D molecular representations, however, do not contain any stereo chemical information, which limits the applications that require the properties depending on internal coordinates or absolute orientation or different conformations. Therefore, we incorporated the 3-D descriptors to represent the 3-D structural features of chemicals. This platform can be used to compute 1053 3-D molecular descriptors. In particular, each molecule will be pre-optimized by MOPAC to obtain the 3D coordinates essential for this calculation. According to the different molecular representations, the molecular descriptors are divided into 20 logical blocks. A list of all molecular descriptors covered by ChemDes is summarized in Table 1. From Table 1, one can see that ChemDes covers a wide range of molecular descriptors and the 2-D descriptors make up most of them. Among all the descriptors, the number of E-state descriptors is obviously more than the others. Moreover, some of the descriptors have been implemented in every toolkit such as the constitutional descriptors, while some others are just implemented in one certain toolkit such as the MoRSE descriptors. This indicates that the structural features are dispersive in these toolkits while the ChemDes provides an integrated approach to access all these molecular descriptors.

**Table 1:** The list of molecular descriptors covered by ChemDes

Type of descriptors	Dimension	Number of descriptors	The origin of features
Constitutional descriptors	1	309	A, B, C, D, E, F
Molecular format descriptors	1	6	A
Autocorrelation descriptors	2	467	C, B, E, F
Basak descriptors	2	63	B, E
BCUT descriptors	2	12	C, E
Burden descriptors	2	160	B, E
Connectivity descriptors	2	194	C, B, D, E, F
E-state descriptors	2	734	B, E
Kappa descriptors	2	92	C, B, E
Molecular property descriptors	2	55	A, B, C, D, E, F
Quantum chemical descriptors	2	7	C, E
Topological descriptors	2	376	B, C, D, E, F
MOE-type descriptors	2	118	B, D
Charge descriptors	2	25	B
3D Autocorrelation descriptors	3	80	E
CPSA descriptors	3	116	B, C, E, F
RDF descriptors	3	390	B, E
Geometrical descriptors	3	62	B, C, E, F
MoRSE descriptors	3	210	B
WHIM descriptors	3	195	B, C, E, F

A, B, C, D, E, F stands for Pybel, Chemopy, CDK, RDKit, PaDEL, and BlueDesc, respectively

### Computation of molecular fingerprints

Molecular fingerprint is a frequently-used abstract representation which allows the computationally efficient handling and comparison of chemical structures. It plays an important role in database search/clustering, similarity screening and molecular diversity analysis. Several toolkits provide a few fingerprinting algorithms, however, these algorithms are dispersive and do not have uniform inputs and outputs sometimes. Besides, it is not easy to use them for users without programming skills. In context of this, ChemDes is developed to compute 59 types of fingerprints without these limitations. Likewise, we have organized and classified the molecular fingerprints. A list of molecular fingerprints covered by ChemDes is summarized in Table 2. The table shows us that plenty of the fingerprinting algorithms are implanted in ChemDes. Each toolkit has its representative implementation of fingerprints, for example, the FP2 fingerprints, FP3 fingerprints from Pybel and the RDK fingerprints from RDKit. At the same time, some fingerprints are covered by different toolkits such as the MACCS fingerprints. It should be noted that the ChemDes platform mainly incorporated jCompoundMapper developed by Hinselmann et al. It provides dozens of popular fingerprinting algorithms for chemical graphs. By providing plenty of fingerprinting algorithms, ChemDes enables users to calculate fingerprints with diversified choices.

**Table 2:** The list of molecular fingerprints covered by ChemDes

Type of molecular fingerprints	The origin of algorithm
FP2 fingerprints	A
FP3 fingerprints	A
FP4 fingerprints	A, B
MACCS fingerprints	A, B, C, D, E, F
Daylight-type fingerprints	B
E-state fingerprints	B, C, D, E
Atom Paris fingerprints	B, D, E
Torsions fingerprints	B, D
Morgan fingerprints	B, D
CDK fingerprints	C, E
Pubchem fingerprints	C, E
CDK extended fingerprints	C, E
Klekota-Roth fingerprints	C, E
GraphOnly fingerprints	C, E
Hybridization fingerprints	C
Substructure fingerprints	C, E
RDK fingerprints	D
Layered fingerprints	D
Pattern fingerprints	D
Klekota-Roth fingerprint count	E
Substructure fingerprint count	E
2D atom pairs count	E
Others^a^	F

A, B, C, D, E, F stands for Pybel, Chemopy, CDK, RDKit, PaDEL, and jCompoundMapper, respectively

^a^Fingerprints from jCompoundMapper: DFS, ASP, AP2D, AT2D, AP3D, AT3D, CATS2D, CATS3D, PHAP2POINT2D, PHAP3POINT2D, PHAP2POINT3D, PHAP3POINT3D, ECFP, ECFPVariant, LSTAR, SHED, RAD2D, RAD3D, MACCS

### Customized computation

In order to make the calculation of molecular descriptors more sophisticated, a customized calculation module is developed. As described above, we have analyzed and classified all the molecular descriptors that ChemDes covers, and then divided these descriptors into several subsets. On the basis of this, we designed and added this module to allow users to calculate certain types of descriptors according to their requirements. This module, firstly, meets the requirements of selecting different types of molecular descriptors to calculate. Secondly, users can also customize different kinds of optimization for 3-D molecular structure information. Thirdly, this module makes it convenient to achieve a study or comparison of the performance of various molecular descriptors. For example, using different types of molecular descriptors with their detailed definitions would be very helpful to variable selection and model explanation when the users establish QSAR models. Besides, it should be an efficient way to save system resources and to make a better user experience that users choose this kind of computation.

### Analysis and discussion

Molecular descriptors can be categorized according to different angles and situations. The main basis that we divide these molecular descriptors into 20 logical blocks is as follows: (a) the elaboration of molecular descriptors from Handbook of Molecular Descriptors [1]; (b) the definition of molecular descriptors from the source code of each toolkit; (c) the definition from the API documentation of each toolkit. In addition, for those descriptors that do not have a clear classification, we categorize some commonly used molecular properties as molecular property descriptors, and categorize some ones that are associated with quantum chemistry as quantum chemical descriptors. Some molecular descriptors that are associated with molecular formats are categorized as molecular format descriptors. The definition and related references for each descriptor are all available in “Library” module mentioned above.

For the purpose of further comparison and study, we retain descriptors that have the same names and come from different toolkits. ChemDes makes it easy to compare the results obtained by different toolkits for the same descriptors. This could be useful when identifying bugs, applying a test suite, or finding the strengths and weaknesses of particular implementations. For example, when different toolkits calculate the same descriptors, it may indicate a bug in one or the other toolkit while the calculated values are not highly correlated.

**Fig. 2:**
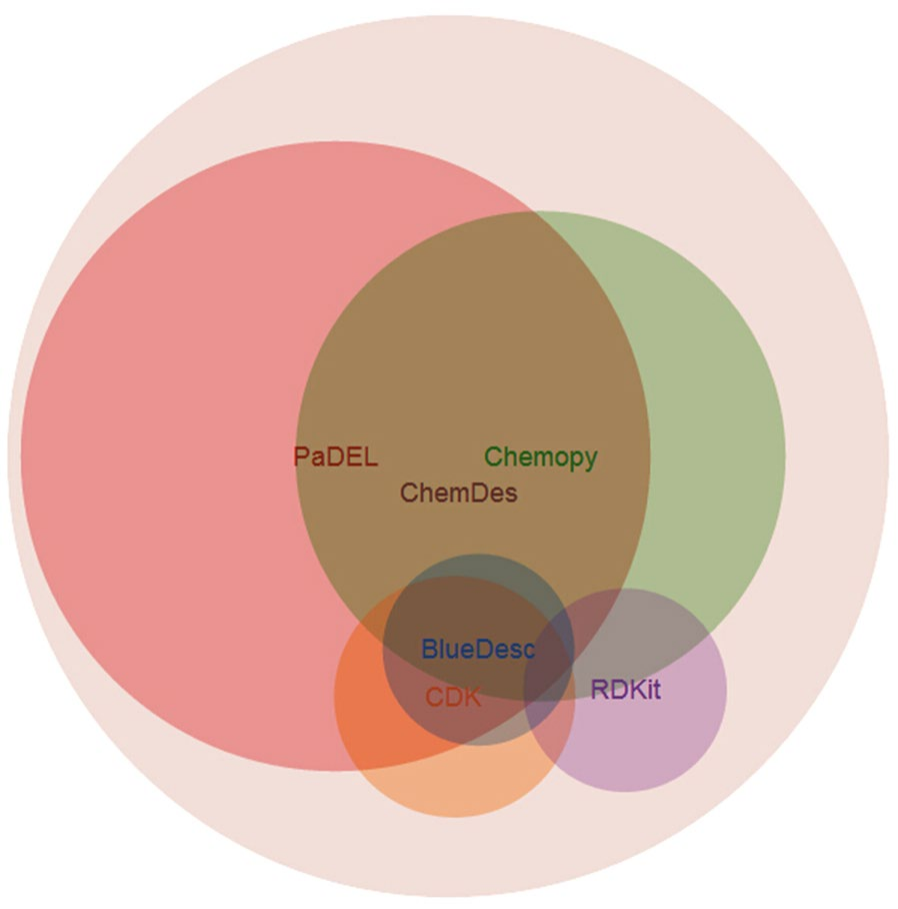
The relationship between the descriptors calculated by different toolkits. The *circles* in different *colors* represent the descriptors from different toolkits. The area size of each *circle* is proportional to the number of descriptors, and the area size of the intersection set is proportional to the number of descriptors they both include

In order to give a clear and meaningful visualization of the relationship between the descriptors covered by ChemDes and those calculated by the other toolkits, the full comparison is performed using a Venn diagram [46], as shown in Fig. 2. The Pybel is not included here, just because the number of descriptor covered by it is too small compared with the others. We consider the descriptors from each toolkit as a set whether there exist ones from other toolkits having the same names with them. The intersection set represents the molecular descriptors two toolkits both include. Of course, there’s one point that should be mentioned: the common descriptors of two toolkits means that they have same names and common basic definitions. But this do not mean that they are totally the same descriptors, because the implementation of the origin algorithms in different toolkits maybe slightly different from each other. For example, the values of “fmf” (a descriptor characterizing complexity of a molecule) separately calculated by PaDEL and CDK are usually not consistent. From Fig. 2, one can clearly see that ChemDes has integrated most of the commonly used toolkits for the calculation of molecular descriptors and fingerprints, and the number of descriptors it covers is also more than the number from other toolkits. Meanwhile, the types of the descriptors are of high diversity. Applying different kinds of molecular descriptors are very necessary for a specific research problem. For example, molecular volume descriptors or those characterizing molecular polarity would be more relevant to study the LogD. As another example, hydrogen bonding related descriptors may be more needed to study drug-target interaction. In the diagram, however, most of toolkits computes several specific molecular descriptors which results in limited types of descriptors. This indicates that descriptors from these toolkits are incomprehensive. Herein, the authors have finally integrated these current toolkits by developing ChemDes to work around this limitation and to give more diversified choices to researchers.

### Features

As described above, we have detailedly presented the ChemDes platform that covers 3679 molecular descriptors with diverse types and 59 types of molecular fingerprints. Compared with the other similar dedicated software instead of with general QSAR software that have descriptor calculation features or programming libraries, ChemDes has several significant advantages: (a) ChemDes is freely available to the public and requires no programming skills. In some cases, molecular descriptor calculation can usually be an important step at the whole project such as QSAR/QSPR, similarity searching, and virtual screening. Researchers just need a freely and easily accessible way to obtain the values, so being free is very helpful. Furthermore, for some pharmacologists and biological scientists, they usually focus more on practical results and data rather than tedious deployment or programming process. Their major focus is rather different from the focus from computational chemistry or chemoinformatics scientists. By using ChemDes they can achieve their goals more rapidly and directly. (b) ChemDes has integrated various molecular descriptors and fingerprints from the toolkits written in different programming languages. As we can see that three types of popular programming languages are used in these toolkits, including C++, Python and Java. On the one hand, limited features represented by a single toolkit will be not so good for users to do a comprehensive comparison and selection. On the other hand, in some cases, it is very difficult or infeasible to restore to a runtime environment with the same configuration of the authors, because most of the toolkits are in form of software or packages which probably have a certain dependence on the operation system and some third-part procedures to a large extent. ChemDes overcomes these problems by accomplishing these complicated tasks on the server side. (c) ChemDes integrates MOPAC software and incorporates three useful tools (ChemCONV, ChemMOP and ChemFPS). ChemDes innovatively combines MOPAC software in a web-based platform to optimize chemical structures. ChemCONV realizes the conversion between various molecular formats conveniently. It allows users to import 7 types of formats and export 11 types for extensive applications, such as *.mop for MOPAC software, *.c3d1 for Chem3D, *.sy2 for Sybyl. ChemMOP supplies geometry and energy information by optimizing molecules using MOPAC. ChemMOP enables users to export 6 types of formats containing 3-D coordinates and provides charge and energy information for wide applications, such as molecular orbital descriptors for the analysis of electron transition in some chemical reactions. ChemFPS provides nine types of similarity measures for users to compare chemical structures. (d) ChemDes possesses advantages of cross-platform and interoperability. Users can access this platform via almost all the operation system types (Microsoft windows, Linux, Mac OS, Android) and client types (PC clients, mobile clients); The calculating results and input/output files from ChemDes can be directly used in other calculations or studies. Of course, such a web-based platform may also have its disadvantages. It’s probably much more difficult to calculate molecular descriptors of a large numbers of chemicals at one time, because a webserver must meet the requirements of a robust system and requests from multiple users. It has been shown, however, that these problems can be overcome by cutting down on the number of chemicals submitted at once and optimizing some related parameters like *timeout* at the back end.

## Conclusions

Considering the amazing rate at which data are accumulated in chemistry and biology fields, new tools that process and interpret large and complex data are increasingly important. The proposed webserver makes a step in this direction providing a way to fully integrate molecular representation information into an easy-to-use web platform. ChemDes provides a convenient and online way to calculate various molecular descriptors and fingerprints. It does not require the time-consuming process of deploying or programming. After representation, different statistical learning tools can be applied for further analysis and visualization of the data. Several studies from different applications show how ChemDes was used to describe various molecular features and establish a model in a routing way. It can be applied to a broad range of scientific fields such as QSAR/SAR, similarity search, absorption, distribution, metabolism, elimination and toxicity (ADMET) prediction, virtual screening, and various interaction data analysis [47]. We expect that ChemDes will better assist chemists, pharmacologists and biologists in characterizing, analyzing, and comparing complex molecular objects.

The current version of ChemDes has a number of strengths that make them useful for a wide variety of applications in chemoinformatics and computational biology. The usefulness of the features covered by ChemDes has been extensively tested by a number of published studies of the development of statistical learning algorithms for analyzing various chemical and biological problems. The similarity principle is prominent in medicinal chemistry, although it is well known as the similarity paradox, i.e., those very minor changes in chemical structure can result in total loss of activity. Based on different similarities, various molecular fingerprint systems were used for identifying novel drug targets. Campillos et al. proposed a novel method to identify new targets based on the similarity of side effects by Daylight-type topological fingerprints. A method to predict protein targets based on chemical similarity of their ligands was proposed by Keiser et al. [18]. using Daylight-type topological fingerprints and extended-connectivity fingerprints. A number of studies have been performed on the modeling of the interaction of GPCR with a diverse set of ligands using a proteochemometrics approach [48, 49], which aims at finding an empirical relation that describes the interaction activities of the biopolymer-molecule pairs as accurately as possible, based on a unified description of the physicochemical properties of the primary amino acid sequences of proteins, and the description of the physicochemical properties of the ligands that may interact with the proteins. The results show that building accurate, robust, and interpretable models for predicting the affinity data is totally possible, provided that suitable representations for proteins and ligands are used.

The main advantages of our proposed webserver are summarized as follows: (1) ChemDes contains a selection of molecular features to analyze, classify, and compare complex molecular objects. They facilitate the exploitation of machine learning techniques to drive hypothesis from complex small molecule datasets, and interaction datasets. The comparative wide coverage of descriptors ensures users to choose the suitable descriptor types relevant to the subject they are studying. (2) ChemDes provides the detailed information about molecular descriptors and how to calculate them in the ‘Library’ and ‘Help’ sections. This helps the researcher to understand the meaning of each descriptor and to interpret the model. (3) ChemDes integrates MOPAC software and incorporates three useful tools (ChemCONV, ChemMOP and ChemFPS). This helps the researchers to apply ChemDes to perform molecular structure optimization, molecular format conversion, and similarity calculation.

Owing to the modular structure of ChemDes, extensions or new functionalities can be implemented easily without complex and time-consuming alterations of the website backstage code. In future work, we plan to apply the integrated features on various biological research questions, and to extend the range of functions with new promising descriptors for the coming versions of ChemDes.

## Availability and requirements

Project name: ChemDes. Project home page: http://www.scbdd.com/chemdes. Operating system(s): Platform independent. Programming language: Python, Java, JavaScript, HTML, CSS. Other requirements: Modern internet browser supporting HTML5 and JavaScript. The recommended browsers: Safari, Firefox, Chrome, IE (Ver. >8). License: MIT. Any restrictions to use by non-academics: None
